# Supplementary material for: Complexome profiling of the Chlamydomonas psb28 mutant reveals TEF5 as an early PSII assembly factor
Source: Plant Cell. 2025 Mar 17;37(6):koaf055. doi: 10.1093/plcell/koaf055 (PMC12164590; doi:10.1093/plcell/koaf055)
Supplement: koaf055_Supplementary_Data [file koaf055_supplementary_data.zip › psb28_interactive.zip!/plots/intensity/qualityPlots/peptides/IDH1_Cre17.g728800.t1.2.html]

### Cre17.g728800.t1.2

Trivial name: IDH1  
SeqCoverage [median]: 33.60  
Mass: 41.08 kDa  
  
 

| Sequence | MissedCleavage | Mass [Da] | wtIntensity | muIntensity | Score |
| --- | --- | --- | --- | --- | --- |
| ENTNTQSLNVQLR | 0 | 1515.76 | 0.00 | 3468.52 | 212.09 |
| ENTNTQSLNVQLRK | 1 | 1643.86 | 515.10 | 0.00 | 28.30 |
| FDYLSGSEETAAGSVPR | 0 | 1784.82 | 1154.22 | 5851.10 | 150.69 |
| FTEAVVR | 0 | 820.44 | 8691.28 | 16017.80 | 143.02 |
| GTLFTPLNK | 0 | 989.55 | 340.02 | 1013.36 | 74.13 |
| SQIVTLIPGDGIGPEVTK | 0 | 1823.00 | 5215.36 | 5573.33 | 147.35 |
| TAQYAFEFAYLNHR | 0 | 1729.82 | 841.60 | 4948.87 | 117.92 |
| TSVPKEVLDSIR | 1 | 1342.75 | 0.00 | 540.09 | 36.30 |
| VITYEK | 0 | 751.41 | 2011.97 | 6679.32 | 97.60 |
| VYTDGDEAALTPDVGGSGTLLR | 0 | 2206.08 | 2494.85 | 4629.28 | 66.05 |
| YNNLDIVVIR | 0 | 1217.68 | 351.67 | 7092.87 | 149.72 |
|  |  |  | 21616.1 | 55814.5 |  |
